# Topical bromfenac in VEGF-driven maculopathies: topical review and meta-analysis

**DOI:** 10.1186/s12886-024-03650-z

**Published:** 2024-08-23

**Authors:** Alexei N. Kulikov, Alexander S. Vasiliev, Yana A. Kalinicheva, Dmitrii S. Maltsev

**Affiliations:** Department of Ophthalmology, Military Medical Academy, 21, Botkinskaya str., St. Petersburg, 194044 Russia

**Keywords:** Bromfenac, Anti-VEGF therapy, Age-related macular degeneration, Diabetic macular edema, Macular edema, Retinal vein occlusion, Non-steroidal anti-inflammatory drugs

## Abstract

**Background:**

Topical non-steroidal anti-inflammatory drugs have the potential to reduce treatment burden and improve outcomes of anti-VEGF therapy for a number of retinal disorders, including neovascular age-related macular degeneration, diabetic macular edema, and retinal vein occlusions. In this review, we focused on the advantages of topical bromfenac as an adjunct to intravitreal anti-VEGF therapy in VEGF-driven maculopathies.

**Methods:**

Cochrane Library, PubMed, and EMBASE were systematically reviewed to identify the relevant studies of neovascular age-related macular degeneration, diabetic macular edema, macular edema associated with retinal vein occlusion, myopic choroidal neovascularization, and radiation maculopathy which reported changes in central retinal thickness, visual acuity, and the number of anti-VEGF injections needed when anti-VEGF therapy was combined with topical bromfenac.

**Results:**

In total, ten studies evaluating bromfenac as an adjunct to anti-VEGF therapy were identified. Five studies were included in meta-analysis of the number of injections and five studies were included in the analysis of changes in central retinal thickness. A statistically significantly lower number of intravitreal injections (*p* = 0.005) was required when bromfenac was used as an adjunct to anti-VEGF therapy compared to anti-VEGF monotherapy with *pro re nata* regimen. At the same time, eyes receiving bromfenac as an adjunct to anti-VEGF therapy demonstrated non-inferior outcomes in central retinal thickness (*p* = 0.07). Except for one study which reported better visual outcomes with combined treatment, no difference in visual acuity or clinically significant adverse effects were reported.

**Conclusions:**

This literature review and meta-analysis showed that topical bromfenac can be considered as a safe adjunct to anti-VEGF therapy with a potential to reduce the treatment burden with anti-VEGF drugs requiring frequent injections without compromising improvement of central retinal thickness or visual acuity.

**Supplementary Information:**

The online version contains supplementary material available at 10.1186/s12886-024-03650-z.

## Introduction

Vascular endothelial growth factor (VEGF) plays a key role in the pathophysiology of many retinal disorders including, but not limited by, neovascular age-related macular degeneration (nAMD), diabetic retinopathy, retinal vein occlusions (RVO) and some entities of the pachychoroid spectrum. The VEGF pathway controls two main pathophysiological mechanisms: neovascularization and vascular leakage [1]. Retinal and choroidal neovascularization are associated with severe and sometimes irreversible vision loss from hemorrhagic complications, and have an impact on long-term outcomes of various retinal diseases [2]. At the same time, leakage can have direct and immediate effects on visual acuity. When occupying the macular area, vascular leakage leads to accumulation of intraretinal and subretinal fluid, which in turn compromises retinal light transduction, function of retinal neurons, causes displacement and alteration of photoreceptors, and dissociation of photoreceptors and retinal pigment epithelium [3]. Today, control of the VEGF pathway through the binding of free VEGF is an important option in the management of many retinal disorders associated with maculopathy [4].

However, VEGF can be considered as a part of a more complex system that includes not only vasculogenic, but also proinflammatory signaling pathways [1, 3]. In this intimate relationship, neovascularization and inflammation may potentiate each other and control permeability of the vascular wall. This agrees with the introduction and propagation of corticosteroids for the management of exudation in diabetic macular edema (DME) and macular edema associated with RVO [5, 6]. Indeed, these drugs simultaneously reduce permeability of retinal vessels and may inhibit the VEGF pathway [7]. However, the latter effect is limited, which is why anti-VEGF drugs are more appropriate for the treatment of the majority of VEGF-driven retinal diseases. At the same time, the opportunity for modifying proinflammatory pathways along with the blocking of VEGF for maximization of treatment outcomes is still not fully incorporated in clinical practice. This is partially related to the high number of intravitreal anti-VEGF injections required during conventional anti-VEGF therapy [8]. Additional injections of corticosteroids would therefore increase the treatment burden. At the same time, current progress in anti-VEGF therapy allows suspension of exudation and regression of neovascularization therefore additional intravitreal therapy with corticosteroids might be excessive. Minimally invasive options facilitating anti-VEGF therapy through anti-inflammatory mechanisms look attractive as a topical rout from both a clinical and financial point of view. However, corticosteroids have relatively high molecular weight and their bioavailability to the posterior eye segment is not sufficient to impact retinal vascular leakage. Alternatively, nonsteroidal anti-inflammatory drugs, such as bromfenac, have a lower molecular weight and reach the posterior eye segment [9, 10]. Although bromfenac cannot be considered as a single treatment option for non-inflammatory maculopathies, it is a useful and safe option, allowing improved management of a number of retinal disorders. In this review, we focused on the potential additional benefits of topical bromfenac as an adjunct to intravitreal anti-VEGF therapy in the most common VEGF-driven maculopathies in changes in central retinal thickness (CRT), visual acuity, and the number of intravitreal injections.

## Methods

A systematic literature review was performed to identify relevant studies published before December 2023focusing on the application of topical bromfenac as an adjunct to anti-VEGF therapy or as monotherapy in VEGF-driven maculopathies. This review and meta-analysis was conducted and reported in adherence to the Preferred Reporting Items for Systematic Reviews and Meta-analysis (PRISMA). The study was not registered, and no protocol was prepared for conducting this study. Electronic databases, including Cochrane Library, PubMed, and EMBASE were searched for keywords, “age-related macular degeneration”, OR “diabetic macular edema”, OR “retinal vein occlusion”, OR “myopic choroidal neovascularization”, OR “radiation maculopathy”, AND “bromfenac”. Additionally, reference list of each identified study was evaluated for the presence of relevant publications. Full-text papers were analyzed if available, otherwise abstracts were evaluated. The following criteria were assessed during analysis: (1) design; (2) condition (nAMD, RVO, or DME, myopic maculopathy, or radiation maculopathy); (3) use of anti-VEGF treatment and name of the drug; (4) treatment in the control group (presence of sham treatment or reference drug); (5) outcomes (number of anti-VEGF injections, best corrected visual acuity (BCVA), and CRT); (6) dosage and duration of bromfenac use; (7) complications, if any. All papers were initially searched by two authors (ASV, YAK) independently followed by assessment by senior researcher (DSM).

Meta-analysis was aimed at assessing the effect of topical bromfenac on CRT and its potential to reduce the treatment burden as an adjunct to anti-VEGF therapy. For this analysis only controlled studies were selected, irrespective of the anti-VEGF drug and treatment regimen. Meta-analysis was performed using MedCalc 18.4.1 (MedCalc Software, Ostend, Belgium) using extracted values for mean, standard deviation, and sample size. Standardized mean difference was used for analysis of the number of anti-VEGF injections and CRT. Heterogeneity was evaluated using the Q-test to calculate the I [2] statistic. Due to substantial heterogeneity among studies the random effect model was adopted. Funnel plots were constructed to display potential publication bias. Sensitivity analysis was performed to assess the consistency of the pooled effect size by excluding one pathology at a time and one study at a time. The Cochrane tool for assessing risk of bias was used. Eight standard points were assessed, including sequence generation, allocation concealment, blinding of participants and personnel, blinding of outcome assessors, incomplete outcome data, selective reporting, and “other bias.” For each bias domain, a “high,” “low” or “unclear” category was assigned. For visual interpretation of the data forest plots were constructed. *P* < 0.05 was considered to be statistically significant.

## Results

In total we identified ten clinical studies on bromfenac as an adjunct to anti-VEGF therapy and two clinical studies which evaluated bromfenac as a monotherapy. All studies used 0.09% or 0.1% topical bromfenac. As an adjunct to anti-VEGF therapy, in six studies bromfenac was used for the treatment of nAMD [11–16], in two studies for DME [17–20], and in two studies for macular edema associated with branch RVO [21, 22]. Two of the ten studies [14, 20] were noncomparative, all others (*n* = 8) having a control group receiving anti-VEGF monotherapy with (*n* = 3) or without (*n* = 5) placebo (Table 1). All studies used *pro re nata* regimen. Decisions regarding the need for subsequent injections after the loading dose, if any, were based on structural optical coherence tomography data.

**Table 1:** Baseline characteristics of the included studies

First author	Year of publication	Condition	Anti-VEGF treatment	Design	Number of participants	Dosage
Grant CA*	2008	nAMD	Ranibizumab	RCT	60	Twice daily
Gomi F*†	2012	nAMD	Ranibizumab	Placebo controlled RCT	38	Twice daily for six months
Flaxel C	2012	nAMD	Ranibizumab	RCT	30	Twice daily for 12 months
Zweifel SA	2009	nAMD	Ranibizumab	Retrospective case series	22	Twice daily for 2 months
Wyględowska-Promieńska D†	2015	nAMD	Aflibercept	RCT	54	Twice daily for 3 months
Wyględowska-Promieńska D*†	2014	nAMD	Bevacizumab	RCT	52	Twice daily for 3 months
Pinna A	2017	DME	-	Prospective case series	17	Twice daily for one months
Gabr AF*†	2023	DME	Ranibizumab	Placebo controlled RCT	70	Twice daily for 6 months
Tobimatsu Y	2023	DME	-	RCT	19	Twice daily for 3 months
Shimura M*†	2015	BRVO	Bevacizumab	Placebo controlled RCT	48	Four times daily for 12 months
Saishin Y	2017	BRVO	Ranibizumab	Placebo controlled RCT	41	Twelve months
Lim B	2023	DME	Bevacizumab	Retrospective case series	14	Twice daily for 3 months

BRVO, branch retinal vein occlusion; DME, diabetic macular edema; nAMD, neovascular age-related macular degeneration; RCT, randomized control trial

*- included in analysis of the number of injections

†- included in analysis of central retinal thickness

In a retrospective case series of Grant, 60 patients received intravitreal ranibizumab for the treatment of nAMD among whom 30 patients received combined treatment with topical bromfenac twice daily for six months. Patients received intravenous fluorescein angiography, optical coherence tomography, and BCVA examination every 4 to 5 weeks over a six-month period. Patients demonstrating activity of nAMD as per optical coherence tomography received additional intravitreal ranibizumab injections. In this study, patients with combined treatment received 1.6 ± 0.69 injections while in the group with intravitreal monotherapy, a mean of 4.5 ± 0.41 injections was performed (*p* = 0.0002).A trend towards better BCVA in the group with combined treatment (р = 0.06) was observed. No adverse effects were described during long-term bromfenac use [11].

Gomi et al. selected a specific cohort of treatment-naive nAMD patients with an occult choroidal neovascularization of less than two-disc diameter. The patients were randomized with a ratio of 2:3 for combined treatment (*n* = 16) or anti-VEGF monotherapy with a sham topical treatment (*n* = 22). Each patient received monthly ophthalmic examination and intravitreal injections of 0.5 mg of ranibizumab in the *pro re nata* regimen. The primary outcome measure was the number of anti-VEGF injections received within six months. Anatomical and functional outcomes were also compared. The mean number of anti-VEGF injections was 2.2 in the group receiving combined treatment and 3.2 in the control group (*p* = 0.027). BCVA showed a higher area under the ROC curve for the entire study period and was numerically higher in all examination points although not statistically significant (*p* = 0.31) in the combined treatment group. CRT trended lower in the group with combined treatment (*p* = 0.06). Multivariable analysis showed that use of topical bromfenac was the only parameter associated with a lower number of ranibizumab injections [12].

Additional benefits from the use of bromfenac as an adjunct to intravitreal ranibizumab in nAMD patients was studied by Flaxel. In this prospective single-center study, 30 nAMD patients were included and randomized with a ratio of 2:1 for combined treatment or anti-VEGF monotherapy. After a loading dose, patients were treated in the *pro re nata* regimen. This study showed no statistically significant difference in BCVA and the number of injections between study groups. However, the mean CRT was statistically significantly lower in the group of combined treatment compared to intravitreal monotherapy, 242.5 and 281.6 μm, respectively (*p* = 0.03). The proportion of eyes with CRT decrease of more than 50 μm was also higher in the group of combined treatment (*p* = 0.046). In general, this was the first prospective study showing a biological signal suggestive of the positive effect of bromfenac as an adjunct to anti-VEGF therapy in nAMD. No safety signals were observed over the 12 month period of bromfenac use [13].

Bromfenac was also studied in a case series of nAMD showing persistent activity despite monthly intravitreal anti-VEGF therapy. In their study, Zweifel and coauthors added topical bromfenac to the anti-VEGF therapy in patients who had received three monthly injections but showed persistent subretinal or intraretinal fluid. BCVA, CRT, and the height of pigment epithelial detachment (PED) were compared at one and two months of combined treatment. Mean BCVA (approximately 0.3 of decimal equivalent) did not change at one (*p* = 0.41) or two months (*p* = 0.26) after initiation of combined treatment. Mean CRT was 311 μm at baseline, 308 μm (*p* = 0.73) at one month and 299 μm (*p* = 0.34) at two months. In 20 eyes with PED, mean high of PED was 275 μm at baseline, 271 μm (*p* = 0.33) and 274 μm (*p* = 0.76) at one and two months of follow-up, respectively. No adverse events were noted during the study period. The limitation of this study was the short follow-up period, retrospective design, and inclusion of treatment-resistant cases [14].

The aim of the study of Wyględowska-Promieńska was to evaluate the efficacy of bevacizumab in combination with bromfenac in nAMD. This was a randomized controlled study including two groups of 26 patients which received three monthly bevacizumab injections followed by *pro re nata* regimen during next three months. Patients in the study group received bromfenac during loading dose twice daily. The authors reported a statistically significant increase of BCVA between 3 and 6 months in the study group but not in the control group. The study group showed higher proportion of eyes with no changes of BCVA, while the proportion of eyes with the loss of one line or more was lower than in the anti-VEGF monotherapy group (*p* = 0.05). No difference was found in the percentage of eyes with improvement of visual acuity. Mean CRT decline was 69 μm in the combined treatment group, but without substantial changes in the control group [15].

In another study of the same authors, bromfenac was studied as an adjunct to aflibercept in the treatment of nAMD. The study included two groups, each of 27 eyes, which received aflibercept monotherapy or aflibercept with topical bromfenac. After the loading dose, additional intravitreal injections were administered based on *pro re nata* regimen. BCVA and anatomical measures were assessed at three and six months after initiation of the treatment. The authors reported statistically significant improvement of BCVA in combined, but not in the monotherapy group. However, the authors did not report if there was any statistically significant difference in baseline BCVA (approximately 0.1 and 0.2 of decimal equivalent) between study groups. There were no statistically significant changes in optical coherence tomography parameters, however combined treatment group showed numerically higher magnitude of the changes. The authors concluded the potential benefits from combined treatment compared to anti-VEGF monotherapy [16].

In the study of Pinna and coauthors, the efficacy and safety of topical bromfenac was evaluated among treatment-naïve DME patients in a prospective case series. In this pilot study, 17 patients with unilateral DME were included. Bromfenac was prescribed to the study eyes for 30 days. Primary outcome measures were changes of BCVA and CRT, however macular volume was also used for analysis. In this study, topical bromfenac significantly reduced mean CRT from 465.4 ± 118.5 μm to 388.9 ± 152.6 μm (р = 0.02). No statistically significant changes of BCVA were found, while decrease of macular volume was close to being statistically significant (р = 0.06). No adverse reactions were reported. The authors concluded that topical bromfenac may play a role in the decrease of DME. However, long-term outcomes remained unknown [17].

Byung-Su Lim also evaluated the short-term results of topical bromfenac in DME. Fourteen eyes of 14 patients diagnosed with DME after a single bevacizumab injection were included. Bromfenac was prescribed for three months. BCVA and CRT were evaluated at baseline and after one, two, and three months. Baseline LogMAR BCVA and CRT were 0.40 ± 0.29 and 337.0 ± 97.3 μm, respectively. LogMAR BCVA improved to 0.39 ± 0.29 at one month, 0.38 ± 0.24 at two months and to 0.34 ± 0.21 at three months, without a statistically significant difference, р = 0.93, р = 0.62, and р = 0.36, respectively. CRT declined statistically significantly from 337 ± 97.3 to 331 ± 67.9 μm at one month, to 311 ± 89.1 μm at two months and to 282.9 ± 76.7 μm at three months, р = 0.47, р = 0.08, and р = 0.04, respectively. No local or systemic adverse reactions were reported [20].

Efficacy and safety of topical bromfenac in DME in a combination with anti-VEGF therapy was studied by Gabr and coauthors. Seventy DME eyes (70 patients) with CRT varying from 300 to 500 μm were included in this study. The patients were randomly selected to combined treatment or to anti-VEGF monotherapy. Both groups received three monthly ranibizumab injections followed by *pro re nata* regimen. From baseline visit each patient received either topical bromfenac or a placebo for the next six months. The groups were compared regarding BCVA, CRT, mean retinal thickness in the macula, and the need for repeated injections. In patients receiving topical bromfenac as an adjunct to intravitreal ranibizumab, statistically significantly better visual outcomes were observed as well as lower CRT, lower mean retinal thickness, and lower need for additional injections compared to the patients receiving ranibizumab only, *p* = 0.013, *p* = 0.010, and *p* = 0.022, respectively. No adverse reactions were register during 6 months of bromfenac use [18].

Effects of topical bromfenac on DME were studied compared to 0.1% betametazone in a controlled study. Nineteen patients with glycosylated hemoglobin level < 8.0% and DME with CRT ranging from 250 to 500 μm were randomized to the monotherapy with bromfenac or betametazone. CRT, BCVA, and IOP were measured after four, eight, and twelve weeks. CRT at baseline (*P* = 0.128) and at every control point showed no statistically significant difference between groups of bromfenac (*n* = 10) and betametazone (*n* = 9). Compared to baseline level, CRT showed a statistically significant decline after eight (*p* = 0.025) and twelve weeks (*p* = 0.043). No changes in BCVA were found in either group. Baseline IOP was comparable between the groups. In the group of betametazone use, IOP level increased at eight (*p* = 0.025) and twelve weeks (*p* = 0.044), however no IOP changes were observed over the entire study period in bromfenac group. In conclusion, bromfenac did not affect IOP as expected, even after twelve weeks of administration and this suggests opportunity for its use in DME patients with a good glycemic control [19].

Efficacy of topical bromfenac in a combination with anti-VEGF therapy was studied by Shimura et al. in eyes with macular edema secondary to branch RVO. The authors included 48 eyes of 44 patients receiving bevacizumab in the *pro re nata* regimen and either topical bromfenac or a placebo. Main outcome measure included CRT and BCVA. The number of injections was also assessed. There were no statistically significant differences in baseline and final CRT or BCVA between eyes receiving bromfenac or placebo. However, the mean number of injections in eyes receiving bromfenac was statistically significantly lower than that in eyes receiving placebo, 3.8 ± 1.1 and 4.8 ± 1.2 injections (*p* < 0.05). The authors concluded that, although bromfenac did not affect functional and anatomical outcomes, it can provide some advantages in terms of reduction of the number of injections. Additionally, in this study with the longest and the most intense bromfenac use among all studies discussed, no adverse reactions relating to the drug were described [21].

Efficacy of bromfenac in combination with ranibizumab for treatment of macular edema associated with branch RVO was also studied in a prospective double blinded placebo-controlled study by Saishin. In this study all patients received one ranibizumab injection followed *pro re nata* regimen with topical bromfenac or placebo. The main outcome measure was the number of intravitreal injections over twelve months. Anatomical and functional outcomes were also evaluated. No difference in baseline BCVA was noted (*p* = 0.26). One year follow-up was completed in 22 patients receiving bromfenac and in 19 patients receiving placebo. The mean number of intravitreal injections was 2.0 in bromfenac group and 3.1 in the placebo group (*p* = 0.032). At the same time, mean BCVA in bromfenac group improved from 0.57 to 0.16 LogMAR (*p* < 0.05), and from 0.56 to 0.15 in the control group (*p* < 0.05). The mean final BCVA was also comparable among study groups (*p* < 0.51). Among eyes receiving bromfenac, the mean CRT declined from 563 μm to 278 μm (*p* < 0.05), and from 618 μm to 250 μm in the control group (*p* < 0.05). The authors therefore concluded that topical bromfenac provides the possibility to reduce of the number of anti-VEGF injections [22].

**Fig. 1:**
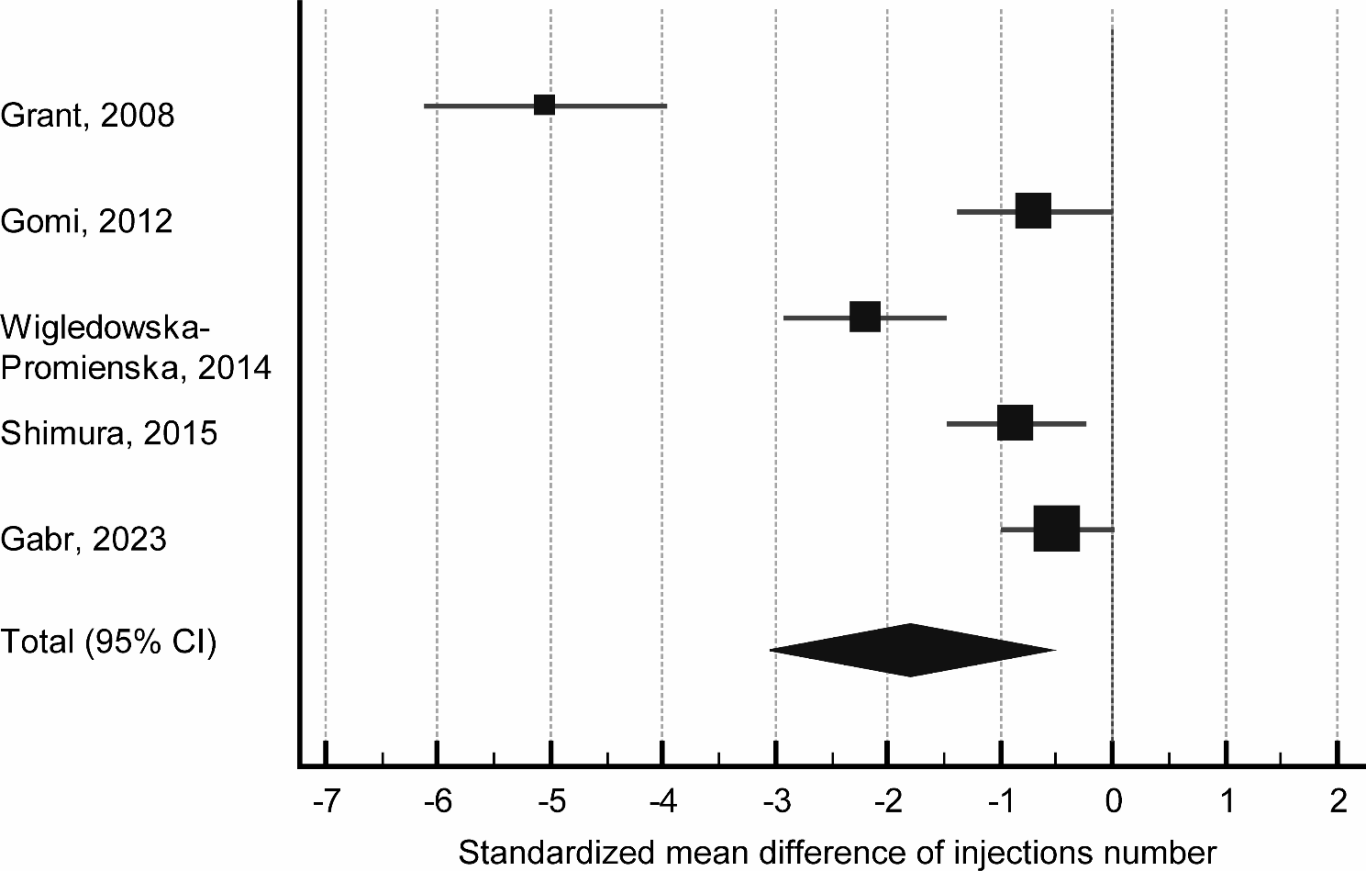
Forest plot showing standardized mean difference of number of anti-VEGF injections comparing combined treatment and anti-VEGF monotherapy

Among the twelve studies selected, analysis of the number of intravitreal injections was unavailable for three papers due to the non-comparative design [14, 17, 20]. Moreover, one paper did not report the number of injections (study of bromfenac in a combination with aflibercept [16]), and two paper did not report standard deviations [13, 22]. Another paper did not evaluate combined treatment [19]. In a study by Gabr and coauthors, the number of injections was not indicated directly, however, it was reported that all patients received three injections, while eight and two patients received additional injection in combined and monotherapy groups respectively [18]. In total, in analysis of the number of injections three nAMD studies, one DME study, and one study of macular edema associated with BRVO were included. Meta-analysis showed a statistically significantly lower number (*p* = 0.005) of intravitreal injections performed when bromfenac was used as an adjunct to anti-VEGF therapy with *pro re nata* regimen (Table 2; Fig. 1).

**Table 2:** Standardized mean difference of number of anti-VEGF injections required in combined treatment and anti-VEGF monotherapy

Study	Combined treatment, *n*	Anti-VEGF monotherapy, *n*	Standardized mean difference	95% CI	*P*-value
Grant, 2008	30	30	-5.043	-6.1 - -4.0	
Gomi, 2012	16	22	-0.689	-1.4 - -0.02	
Wigledowska-Promienska, 2014	26	26	-2.200	-2.9 - -1.5	
Shimura, 2015	24	24	-0.854	-1.45 - -0.26	
Gabr, 2023	35	35	-0.483	-1.0 - -0.004	
Total (random effects model)	131	137	-1.614	-3.1 - -0.11	0.005

Heterogeneity: Q = 74.2.6; df = 4 (*P* < 0.001); I^2^ = 94.6% (95% CI for I^2^ = 90.2 to 97.0)

**Fig. 2:**
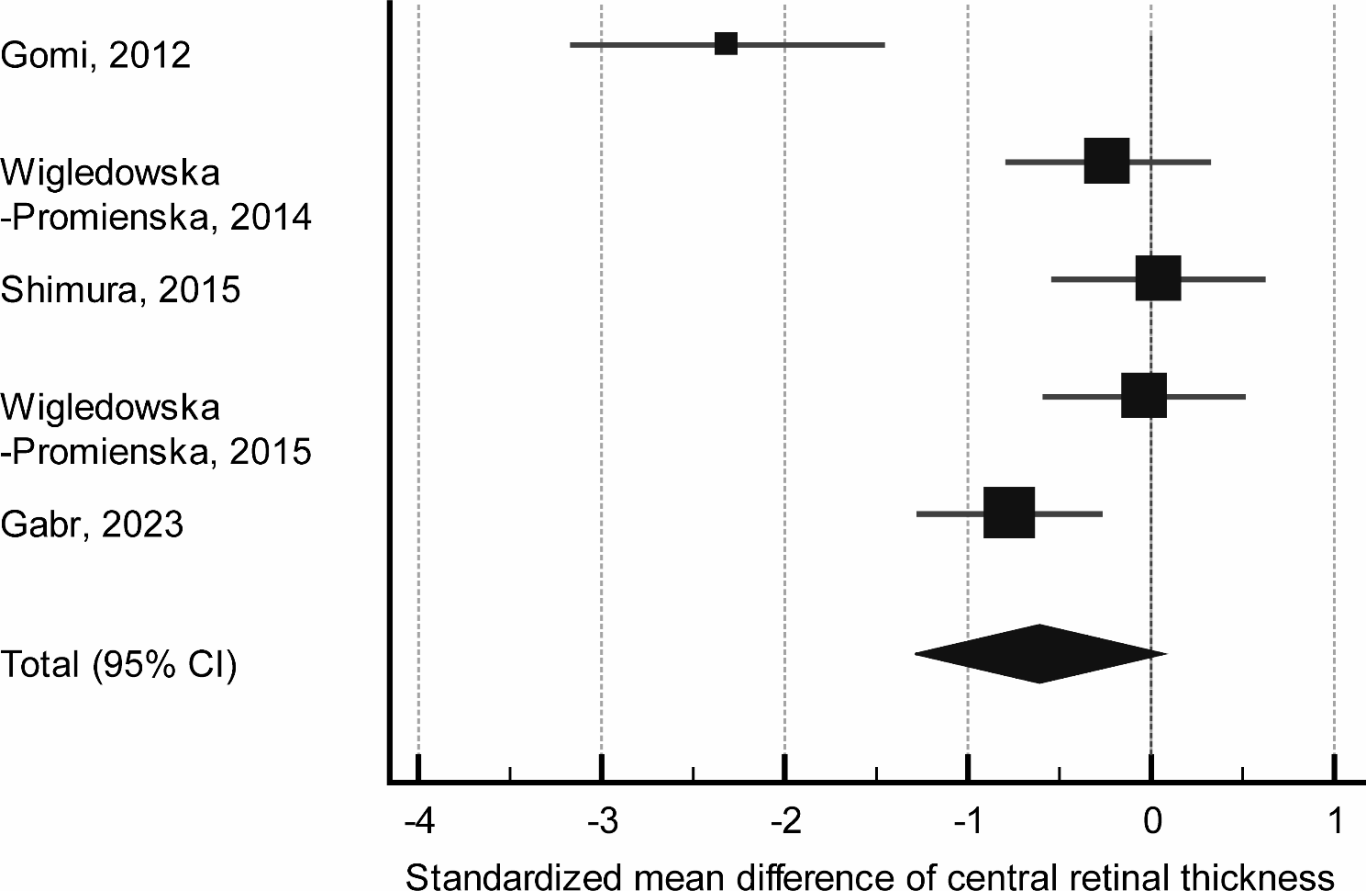
Forest plot showing standardized mean difference of central retinal thickness comparing combined treatment and anti-VEGF monotherapy

Among the twelve studies selected, analysis of CRT was impossible in the three non-comparative studies [14, 17, 20], in two studies which did not report exact CRT values [11, 22], and in one study where standard deviations were not reported [13]. Therefore, meta-analysis included three papers which studied nAMD, one paper studying DME, and one focusing on BRVO. This meta-analysis showed non-inferior CRT outcomes (*p* = 0.07), with numerically lower values, in eyes receiving bromfenac as an adjunct to anti-VEGF therapy (Table 3; Fig. 2).

**Table 3:** Standardized mean difference of central retinal thickness in combined treatment and anti-VEGF monotherapy

Study	Combined treatment, *n*	Anti-VEGF monotherapy, *n*	Standardized mean difference	95% CI	*P*-value
Gomi, 2012	16	22	-2.317	-3.2 - -1.5	
Wigledowska-Promienska, 2014	26	26	-0.234	-0.8–0.32	
Shimura, 2015	24	24	0.0439	-0.5–0.6	
Wigledowska-Promienska, 2015	27	27	-0.0354	-0.58–0.5	
Gabr, 2023	35	35	-0.772	-1.3 - -0.3	
Total (random effects model)	128	134	-0.6	-1.3 - -0.06	0.07

Heterogeneity: Q = 27.7; df = 4 (*P* < 0.001); I^2^ = 85.6% (CI 95% for I [2] 68.2 to 93.5)

In this study we did not evaluate the benefits of combined treatment on BCVA since only one out of twelve studies reported visual improvement with the additional use of bromfenac, while others showed no statistically significant difference between combined treatment and monotherapy. No specific adverse events associated with bromfenac use or the difference between combined and control group were reported among twelve studies in terms of safety signals, except in one case where “unpleasant sensation” led to bromfenac treatment discontinuation [12].

Our sensitivity analysis showed that nAMD studies are mostly responsible for the high heterogeneity in the analysis of the injection numbers, however no individual nAMD study makes a particular impact on the heterogeneity (I^2^ 82.7–95.8%). In the analysis of CRT, no particular condition specifically affects heterogeneity (I^2^ 78.9–87.3%). Among all studies, the results of Gomi et al. substantially affect heterogeneity (without this study I [2] reduced to 51.5%) of CRT analysis probably due to inclusion of mild nAMD cases resulting in low final CRT. Funnel plots showed that there is a risk for potential publication bias mostly related to study of Grant et al. [11] in analysis of the number of injections and study of Gomi et al. [12] in analysis of CRT changes (Supplementary figure). Assessment of the risk of bias showed, in general low risks for the studies included (Table 4).

**Table 4:** Assessment of risk of bias of the included studies

	Grant et al. 2008	Gomi et al. 2012	Wyględowska-Promieńska et al., 2014	Wyględowska-Promieńska et al. 2015	Shimua et al. 2015	Gabr et al. 2023
Random sequence generation	High risk	Low risk	High risk	High risk	Low risk	Low risk
Allocation concealment	High risk	Unclear	High risk	High risk	Low risk	Low risk
Blinding for the outcomes	Low risk	Low risk	Low risk	Low risk	Low risk	Low risk
Blinding of participants and personnel	High risk	Low risk	High risk	High risk	Low risk	Low risk
Blinding of outcome assessors	Low risk	Low risk	Low risk	Low risk	Low risk	Low risk
Incomplete outcome data	Low risk	Low risk	Low risk	Low risk	Low risk	Low risk
Selective reporting	Low risk	Low risk	Low risk	Low risk	Low risk	Low risk
Other bias	Low risk	Low risk	Low risk	Low risk	Low risk	Low risk

## Discussion

This review revealed a number of peer-reviewed papers focusing on bromfenac as an adjunct to anti-VEGF therapy in neovascular age-related macular degeneration, diabetic macular edema, and macular edema associated with retinal vein occlusion. Comparative studies indicate an opportunity to reduce the number of anti-VEGF injections with combined treatment, without compromising anatomical and functional results.

All studies included in the review used 0.09% or 0.1% formulation of bromfenac. The duration of bromfenac use varied from one to twelve months, with two to four drops administered daily. Despite prolonged use of bromfenac, up to twelve months, the combined treatment was not associated with any adverse effects.

In the majority of studies, bromfenac was used in combination with anti-VEGF drugs which required frequent injections. The current progress in anti-VEGF therapy is aimed on the reduction of treatment burden and resulted in introduction of new anti-VEGF drugs including brolucizumab and faricimab [23, 24]. However, the role of early generation drugs such as ranibizumab and aflibercept remains considerable. Reduction of the treatment burden is therefore still significant for clinicians and moreover is a question of cost-efficacy. It is also worth noting, that topical bromfenac significantly reduces the pain immediately after and six hours postintravitreal injection [25]. Therefore, its use in patients receiving regular injections may reduce discomfort associated with the procedure and fear of the injection.

The difference in CRT between combined treatment and monotherapy did not reach statistical significance, however with a trend toward numerically lower CRT in combined treatment. On the other hand, absence of substantial difference in CRT is to be expected since during adequate anti-VEGF treatment CRT reaches low values.

Interestingly, a few studies analyzed the results of bromfenac monotherapy in DME [17, 19]. This approach looks viable for this condition since proinflammatory pathways in DME play an important role (probably the most significant compared to other VEGF-driven maculopathies) and the cases selected for this monotherapy were limited by mild to moderate DME in terms of CRT. These studies showed anatomical improvement without any side effects.

The limitations of this review include the combining of different retinal pathologies for meta-analysis. Although the role of inflammation differs between nAMD, DME, and retinal vein occlusions, all of them were included in the analysis since all of them requires anti-VEGF therapy and are associated with the treatment burden. However, the sensitivity analysis has shown that exclusion of any specific pathology does not substantially affect the heterogeneity in analysis neither CRT nor number of injections. Another limitation is the difference in bromfenac dosage and duration of use between different studies, although this highlights the safety of topical bromfenac. Finally, funnel plots analysis showed that potential publication bias may be associated with study of Grant et al. in analysis of the number of injection and study of Gomi et al. [12] in analysis of CRT changes (Supplementary figure). The study Gomi et al. in contrast to others, included mild nAMD with small lesions, which may explain the low compared to other studies values of CRT. In the study of Grant et al. the difference in the number of injections between combined treatment and control group was substantially higher than in other studies (approximately three injections versus one) which remained unexplained by the authors.

In conclusion, this literature review and meta-analysis showed that topical bromfenac can be considered as a safe adjunct to anti-VEGF therapy which may help to reduce the treatment burden without compromising visual acuity or central retinal thickness when drugs requiring frequent injections are used in *pro re nata* regimen or where fixed dose anti-VEGF therapy looks redundant.

### Electronic supplementary material

Below is the link to the electronic supplementary material.

Supplementary Material 1

## Data Availability

No datasets were generated or analysed during the current study.
