# Supplementary figures and images for: Topical bromfenac in VEGF-driven maculopathies: topical review and meta-analysis
Source: BMC Ophthalmol. 2024 Aug 23;24:369. doi: 10.1186/s12886-024-03650-z (PMC11344392; doi:10.1186/s12886-024-03650-z)

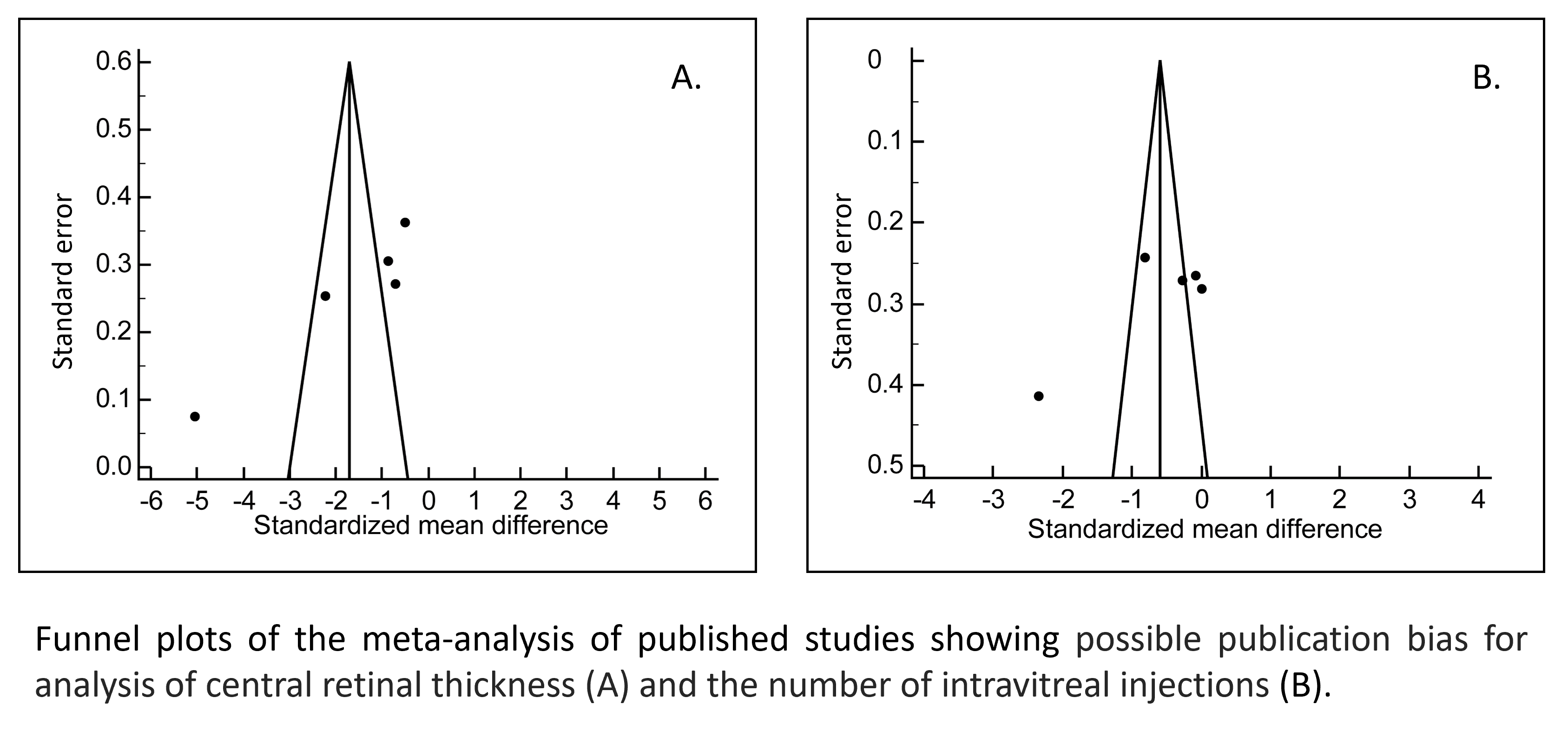

Supplement: Supplementary file 1 — Supplementary Material 1 [file 12886_2024_3650_MOESM1_ESM.png]
